# A role for the histone chaperone DAXX in the structural organization of heterochromatin

**DOI:** 10.1186/1756-8935-6-S1-P121

**Published:** 2013-04-08

**Authors:** Lindsy M Rapkin, Kashif Ahmed, Ren Li, David P Bazett-Jones

**Affiliations:** 1 Program in Genetics and Genome Biology, The Hospital for Sick Children, Toronto, Ontario, Canada, M5G 1 L7; 2 Department of Biochemistry, University of Toronto, Toronto, Ontario, Canada, M5S 1A8

## Background

Originally described as a cytoplasmic protein involved in Fas-mediated cell death, the death-domain associated protein (DAXX) is predominantly localized in the nucleus where it functions as a transcription repressor and a mediator of apoptosis. Recently, DAXX has been identified as a histone chaperone, where together with the ATP-dependent chromatin remodeling factor ATRX, it deposits the histone variant H3.3 at telomeres and in regions of pericentric heterochromatin in mouse embryonic stem cells and fibroblasts, respectively [1,2]. DAXX has been shown to associate with and regulate the transcription of major satellite repeats. This, together with the requirement for H3.3 deposition for heterochromatin formation in early mouse development [3], underscores the importance of gaining insight into the role of DAXX in the structural organization of heterochromatin.

## Materials and methods

We utilized established wild type and DAXX null fibroblast cell lines and a combination of imaging-based techniques including immunofluorescence, fluorescence in situ hybridization (FISH), and correlative electron spectroscopic imaging (ESI), a specialized form of energy-loss transmission electron microscopy. ESI allows us to visualize element-specific distributions with high spatial resolution. In particular, phosphorus and nitrogen atomic imaging can be used to distinguish the nucleic acid from protein-based structures within a biological sample. Furthermore, specific nuclear structures can be targeted by virtue of their inherent biochemical properties.

## Results

We show that H3K9me3-enriched heterochromatin domains (chromocentres) are disrupted in the absence of DAXX. Unlike chromocentres from wild type cells, which are radially symmetric, compact, and phosphorus-rich, those from DAXX null cells are often irregularly shaped with altered phosphorus to nitrogen ratios and these abnormal chromocentres make unusual contacts with the surrounding chromatin. Immuno-FISH experiments demonstrated an aberrant spatial relationship between H3K9me3-enriched structures and major satellite DNA. Additionally, we show that the loss of DAXX results in disorganized nucleoli and ribosomal DNA (rDNA), and an increased number of cells containing mini nucleoli.

## Conclusions

These findings demonstrate that loss of the histone chaperone DAXX results in the uncoupling of an epigenetic mark from the underlying chromatin structure and identifies a novel role of DAXX in the structural organization of heterochromatin.
